# Turkish Final Year Medical Students’ Exposure to and Attitudes Concerning Drug Company Interactions: A Perspective from a Minimally Regulated Environment for Medical Students

**DOI:** 10.1371/journal.pone.0168094

**Published:** 2016-12-15

**Authors:** Nazim Ercument Beyhun, Cevriye Ceyda Kolayli, Gamze Can, Murat Topbas

**Affiliations:** Karadeniz Technical University Medical Faculty Dep. Of Public Health, Trabzon, Turkey; York University, CANADA

## Abstract

Interactions between drug companies and medical students may affect evidence-based medical practice and patient safety. The aim of this study was to assess drug company–medical student interactions in a medical faculty where limited specific national or institutional regulations apply between drug companies and medical students. The objectives of the study were to determine the exposure and attitudes of final year medical students in terms of drug company–medical student and physician interactions, to identify factors affecting those attitudes and to provide data for policymakers working on the regulation of interactions between drug companies and medical students. This anonymous questionnaire-based study of 154 medical final year medical students at the Karadeniz Technical University Medical Faculty, Trabzon, Turkey, in April and May 2015 attracted a response rate of 92.2% (n/N, 154/164). Exposure to interaction with a pharmaceutical representative was reported by 90.3% (139/154) of students, and 68.8% (106/154) reported experiencing such interaction alongside a resident. In addition, 83.7% (128/153) of students reported an interaction during internship. Furthermore, 69.9% (107/153) of students agreed that interactions influence physicians’ prescription preferences, while 33.1% (51/154) thought that a medical student should never accept a gift from a drug company and 24.7% (38/154) agreed with the proposition that “drug companies should not hold activities in medical faculties”. Students with rational prescription training expressed greater agreement with the statement “I am skeptical concerning the information provided by drug companies during interactions” than those who had not received such training, and this finding was supported by logistic regression [O.R.(C.I), p -3.7(1.2–11.5), p = 0.022]. Acceptance of advertisement brochures was found to significantly reduce the level of agreement with the proposition that “A physician should not accept any gift from a drug company.” (0.3[0.1–0.9], p = 0.030). In summary, exposure to drug companies was widespread among our final year medical students who, like students in both Western and non-Western societies, hold permissive attitudes concerning accepting gifts, and drug advertising brochures may relax those permissive attitudes still further. Rational prescription training was useful in generating rational attitudes. Policies concerning drug company–medical student relationships should be developed in Turkey as well as internationally.

## Introduction

Interactions between the drug industry and physicians are a major subject of concern in the medical world [1, 2]. Research has shown that exposure to drug companies influences physicians’ prescription choices [3–9]. Drug company—physician interactions have been shown to cause non-rational prescription choices, such as prescribing drugs with no significant superiority over others, more expensive drugs and fewer generic drugs [7]. Medical students are also targeted by the drug industry and participate in various drug company-sponsored activities from their first year in medical school. Contact between drug companies and students starts in classes and continues in clinical training [1]. Students’ exposure to and attitudes concerning drug companies during clinical training, when prescription preferences and habits are acquired, are therefore highly important.

Potential adverse effects and biases arising during drug company–student interactions can be reduced through regulations restricting drug company-trainee contacts, learning about evidence-based medicine and acquiring rational prescribing behaviors and habits [3, 10–13]. It is therefore very important that faculties of medicine should develop and apply conflict of interest (COI) policies and monitor their results. The PharmFree scorecard was launched by AMSA (American Medical Student Association) in 2007 and has been updated every year in order to provide an assessment of the strength of policies regarding interactions between drug companies and students at faculties of medicine in the United States of America (USA). This scorecard makes a significant contribution to the assessment of COI policies of medical faculties, as well as indicating the degree of transparency of interactions between faculties and drug companies and thus obliging them to regulate those interactions in the USA [14]. In 2010, Mathieu et al. identified deficiencies in respect of COI policies by applying the AMSA scorecard to 16 faculties of medicine in Canada. This was supported by a similar study by Shnier et al. in 2013 which showed that faculties of medicine in Canada were still permissive in respect of the COI policy environment [15, 16]. In France, interactions between drug companies and students were regulated by law at the end of 2011. Etain et al. subsequently determined that students at faculties of medicine in France possessed insufficient knowledge of COIs [17].

The directive concerned with drug company interactions in Turkey is Regulation No. 29405, published in the Official Gazette on 3 July, 2015 [18]. This regulation contains two articles referring to medical students. Under Article 5, students are permitted to take part in activities arranged by drug companies. Article 6 states that in addition to a list of names of participating physicians, the names of medical students involved in these interactions must also be reported to the Medical Devices and Drug Institution. This regulation covers drug company interactions held in health institutions and relations between physicians and companies. It imposes a number of restrictions on physician-company relations, but makes no specific regulations for students. These regulations will inevitably affect students to some extent, even if they make no specific provisions for them. In addition, the universities at which the research was performed have no policies for regulating interactions between drug companies and students.

Most studies of drug company-physician and drug company-student interactions have been conducted in the developed world [19]. More recently, however, there have been many studies of exposure on the part of physicians and medical students to drug company interactions in the developing world. Pakistani, Kuwaiti and Nepalese medical students are commonly exposed to drug company interactions, which may well have an influence on them [20–22]. In addition to these studies of medical students, there have also been numerous studies of drug company—physician interactions in other developing countries. Researchers from Libya, Egypt, Peru and Saudi Arabia have reported that accepting gifts and other promotional materials and attending drug company sponsored meetings are widespread, and many physicians have reported that they might be influenced by pharmaceutical representatives [23–26].

The only previous study from Turkey concerned the exposure of second and third (preclinical) year medical students to drug companies and their observations of drug company–physician interactions in primary health care settings between 2003 and 2006. That study concluded that pharmaceutical representatives taking a specific interest in students resulted in students adopting more positive opinions toward drug company interactions [27]. Ours is the first Turkish study of final year medical students (sixth year medical students) completing their clinical training immediately prior to graduation.

Medical training in faculties of medicine in Turkey lasts six years. Students spend their first three years on preclinical studies. From the fourth year, students embark on clinical training. In the sixth (final) year, in which this research was conducted, supervised medical practice is undertaken in the form of internships. During internships, all students receive rational prescription training.

### Rational prescription training

All students in our faculty have to undergo rational prescription training during their internships (sixth year of medical training). This lasts one week and involves the participation of approximately 30 students on a periodic basis (once every two months) during the year. During this training, students are given prepared cases for which they then write prescriptions. Additionally, all final year medical students encounter simulated patients with different diseases for whom they write prescriptions using the generic names of drugs. Brand names are not used or discussed during this process. Students’ written prescriptions are then discussed in terms of medical suitability and cost analysis, as well with professors from the Department of Pharmacology. Students also attend a lecture in pharmacoeconomics discussing the global and Turkish drug markets, and drug pricing is discussed. At the time of this study, some students had received that training and others had not. This made it possible to determine the effect of training on attitudes.

The purpose of this research was to determine the extent of exposure to drug companies, the attitudes concerning drug company interactions among final year medical students, to determine whether exposure to drug companies (exposure to drug company representatives or receiving promotional products such as drug advertisement brochures, meals, small gifts, free drug samples, or sponsored travel) and prior rational prescription training affects their attitudes and to provide data for policy makers working on the regulation of interactions between drug companies and medical students in the short and long terms.

## Methods

### Sampling and data collection

The research data were collected during April and May, 2015, using a questionnaire prepared by the authors employed for the first time in this study. The students completed the printed questionnaires by themselves. We aimed to reach all students (N = 164). All our students were invited to participate, and were visited three times. The questionnaires were distributed on all three visits, and students were asked to complete them on all visits.

### The questionnaire

The questionnaire consisted of three sections. The first contained questions concerning the characteristics of the students (age, gender, time left to graduation, having a family member in the drug industry, intention to work in the industry after graduation, prior rational prescription training). The second inquired into exposure to drug company interactions and the extent of that exposure (exposure to drug company representatives, exposure during internship, in which grade exposure first occurred, where the first exposure occurred) and receipt of drug company sponsored products such as drug advertisement brochures, meals, non-educational small gifts, free drug samples, textbooks. The third section investigated the students’ attitudes toward interactions between drug companies and physicians or students.

### Propositions regarding attitudes

Nineteen propositions were evaluated in three separate parts:

1. The influence of drug companies on prescription writing and students’ knowledge of drugs
2. Skepticism toward accepting drug sponsored gifts and products
3. Skepticism toward interactions between drug companies and students or physician (other than accepting gifts)

Students then cited levels of agreement or disagreement (strongly disagree-disagree-undecided-agree-strongly agree) with the propositions expressed. The translated questionnaire is attached as an appendix.

### Analysis pathway

1. The characteristics and the extent of students’ exposure to drug company interactions were evaluated using descriptive statistics such as numbers, percentages, means and standard deviations.
2. The distribution of students’ attitudes was analyzed using numbers and percentages.
3. Analysis of factors affecting students’ attitudes

These factors were a; Exposure to drug company representatives (exposure / no exposure), b; Exposure during internship (exposure / no exposure), c; Prior rational prescription training (yes / no), and d, Acceptance of drug company sponsored gifts and products (drug advertisement brochures, small, non-educational gifts, meals, free drug samples, textbooks) (never / at least once)

### Determining factors

Responses towards propositions were dichotomized into agreement with each statement (strong agreement or agreement) or disagreement and indecision (strong disagreement or disagreement or indecision). Acceptance of drug company sponsored gifts and products (drug advertisement brochures, small, non-educational gifts, meals, free drug samples, textbooks) was quantified as ‘never’ or ‘accepted at least once’ in order to maintain adequate numbers of observations for statistical analysis.

We first used Pearson’s chi square test and Fisher’s Exact test to test the relationship between factors [exposure of students to drug company representatives, exposure during internship, prior rational prescription training, and acceptance of drug company sponsored gifts and products (drug advertisement brochures, small, non-educational gifts, meals, free drug samples, textbooks)] in univariate analysis. Multiple logistic regression was then used to determine factors related to students’ attitudes. At multiple logistic regression, the dependent variables identified were the attitudes of students. Control variables were time remaining to graduation, having a family member in the drug industry, and intention to work in the industry after graduation. Independent variables were exposure to drug company representatives, exposure to drug company interactions during internship, prior rational prescription training and acceptance of drug company sponsored gifts and products (drug advertisement brochures, small, non-educational gifts, meals, free drug samples, textbooks). Odds ratios (OR) in the models were calculated as the affecting coefficient for exhibition of the relevant attitude. P values less than 0.05 were regarded as statistically significant. Statistical analyses were performed on SPSS 13.0 (Chicago, USA) software.

### Ethics

Participants were informed about the study in a written summary at the top of the questionnaire, which stated the aim of the study and indicated that participation constituted consent. The students filled in the questionnaires anonymously. Ethical committee approval is not obligatory in Turkey for non-clinical studies based on anonymous questionnaires. The research was approved by the Karadeniz Technical University Faculty of Medicine Board of Directors.

## Results

### Response Rate

Of the 164 students invited to participate, 154 responded (92.2%). All the questionnaires were included in the study.

### 1. Characteristics of the students

Mean age of the students was 25.1±1.2 years (n = 152), and 56.5% (87/154) were women. Mean time to graduation at the time of the study was 3.7±2.1 months (n = 150). The questionnaire revealed that 18.8% (29/154) of respondents were related to or well acquainted with a pharmaceutical representative and 5.3% (8/152) were planning a career with a drug company. Of the respondents, 69.7% (99/142) reported that they had received rational prescription training during their education. Being related to, or well acquainted with a pharmaceutical representative or wishing to make a career with a drug company did not affect the attitudes of students in univariate analysis but being related to or well acquainted with a pharmaceutical representative was associated with the attitude “I think that drug companies should support institutions rather than supporting physicians” in logistic regression (p>0.05 for other attitudes). Being related to or well acquainted with a pharmaceutical representative increased agreement with this proposition.

### 2. Exposure of students to drug company interactions and the extent of such exposure

Exposure to interaction with a pharmaceutical representative throughout subjects’ entire medical education was reported by 90.3% (139/154) of students, and 68.8% (106/154) reported experiencing such interaction alongside a resident. Moreover, 83.7% (128/153) of students reported an interaction during their internship (6^th^ year). Only four (2.6%) students report no form of drug company exposure. Details of interactions and the extent of the exposures involved are presented in Table 1.

**Table 1:** Students’ exposure to drug company interactions and the extent of that exposure.

Characteristics of exposure	n	%
**Exposure to drug company representatives** (N = 154)		
Yes	139	90.3
Alongside a resident	106	68.8
Alongside an instructor physician	38	24.7
In a student group	35	22.7
One-to-one	30	19.5
**Exposure to drug company interactions during internship** (N = 153)		
Exposure	128	83.7
No exposure	25	16.3
**Class of first exposure to drug company interaction** (N = 144)		
1	2	1.4
2	3	2.1
3	12	8.3
4	50	34.7
5	45	31.3
6	32	22.2
**Site of first drug company exposure** (N = 146)		
Lecture room	24	16.4
Scientific congress	5	3.4
Meal	9	6.2
Out-patient clinic	72	49.3
In-patient clinic	26	17.8
Other	10	6.8
**Acceptance of advertisement brochures** (N = 142)		
Never	34	23.9
Once	19	13.4
2–5 times	59	41.5
More than 5 times	30	21.1
**Acceptance of small, non-educational gifts** (N = 147)		
Never	28	19.0
Once	38	25.9
2–5 times	70	47.6
More than 5 times	11	7.5
**Acceptance of free drug samples** (N = 138)		
Never	84	60.9
Once	32	23.2
2–5 times	20	14.5
More than 5 times	2	1.4
**Acceptance of textbooks** (N = 135)		
Never	119	88.1
Once	11	8.1
2–5 times	5	3.7
More than 5 times	-	-
**Acceptance of drug company sponsored scientific travel to congress** (N = 136)		
Never	133	97.8
Once	2	1.5
2–5 times	1	0.7
More than 5 times	-	-
**Acceptance of meals sponsored by drug companies** (N = 136)		
Never	84	61.8
Once	26	19.1
2–5 times	25	18.4
More than 5 times	1	0.7

### 3. Attitudes of students toward interactions between drug companies and medical students or physicians

Students’ attitudes toward interactions between drug companies and medical students or physicians are shown in Table 2.

**Table 2:** Attitudes of students towards interactions between drug companies and medical students or physicians.

		Strongly disagree	Disagree	Undecided	Agree	Strongly agree
		%	%	%	%	%
**Influence of drug companies on prescription and perception of own knowledge of drugs**						
P5	Interactions influence physicians’ prescription preferences.(n = 153)	5.2	12.4	12.4	64.7	5.2
P7	I think that interactions influence resident physicians’ prescription preferences. (n = 154)	5.2	13.6	13.0	60.4	7.8
P16	The interactions that I was exposed to may influence my future prescription preferences. (n = 154)	9.7	24.7	15.6	48.7	1.3
P1	I possess sufficient knowledge about drugs to function as a general practitioner. (n = 154)	11.7	53.9	3.9	28.6	1.9
P14	My level of medical knowledge is sufficient to assess the information in drug advertisement brochures. (n = 153)	6.5	26.1	7.2	49.0	11.1
**Skepticism towards accepting drug sponsored gifts and products**						
P6	A public employee should never accept gifts. (n = 152)	4.6	23.0	14.5	37.5	20.4
P9	A medical student should never accept a gift from a drug company. (n = 154)	13.0	38.3	15.6	24.7	8.4
P11	There is nothing wrong in accepting small gifts as reminders, such as pens, key rings, memory sticks or bags. (n = 154)	12.3	19.5	11.0	45.5	11.7
P13	A physician should not accept any gift from a drug company. (n = 154)	11.7	31.2	15.6	28.6	13.0
P15	I see nothing wrong in physicians attending scientific meetings sponsored by drug companies. (n = 154)	7.1	12.3	7.8	62.3	10.4
**Skepticism towards drug company–students–physician interactions other than accepting gifts**						
P2	Interactions are an important source of information. (n = 154)	7.8	41.6	13.6	35.7	1.3
P3	I trust the information in drug advertisement brochures. (n = 152)	10.5	53.3	19.1	17.1	-
P4	The information provided during interactions is impartial. (n = 152)	30.9	51.3	7.2	8.6	2.0
P8	Interactions with students need to be subjected to legal regulation. (n = 153)	4.6	9.2	12.4	59.5	14.4
P10	I think that interactions with students are inadequate and need to be increased. (n = 151)	4.0	18.5	19.2	47.7	10.6
P12	Drug companies should not hold activities in medical faculties. (n = 154)	7.8	53.9	13.6	14.9	9.7
P17	I regard interactions between pharmaceutical representatives and physicians as proper. (n = 154)	14.3	20.1	20.8	42.9	1.9
P18	I am skeptical concerning the information provided by drug companies during interactions. (n = 153)	2.0	11.1	5.9	64.7	16.3
P19	I think that drug companies should support institutions rather than supporting physicians. (n = 153)	3.3	20.3	27.5	35.3	13.7

#### a. Influence of drug companies on prescription writing and perception of students’ own knowledge of drugs

In this study, 69.9% (107/153) and 68.2%(105/154) of students agreed that the interactions influence physicians’ or resident physicians’ prescription preferences, respectively, However, only 50.0% (77/154) agreed that their future prescription writing behavior might be affected. Those students who agreed that physicians are affected also exhibited significantly higher agreement that their own future prescription writing behavior might be affected (60.7 vs 23.9%, p<0.001).

#### b. Skepticism toward accepting drug company sponsored gifts and products

In this research, 33.1% (51/154) of students agreed that a medical student should never accept any gift from a drug company. However, 57.1% (88/154) and 72.7% (112/154) of students saw nothing wrong in accepting small gifts or attending drug company sponsored scientific meetings, respectively.

#### c. Skepticism toward interactions between drug companies and students or physicians other than acceptance of gifts

Of the respondents, 37.0% (57/154) agreed that “Interactions are an important source of information.” However, only 10.6% (16/152) agreed or strongly agreed that “The information provided during interactions is impartial.” A further 73.9% (113/154) of students agreed that interactions with students need to be subjected to legal regulation. Finally, 81.0% (124/153) of students agreed that they were skeptical concerning the information provided by drug companies during interactions.

### 4. Factors affecting students’ attitudes

The associations between factors and attitudes of students are shown in Table 3 (exposure to drug company representatives, exposure to drug company interactions during internship and prior rational prescription training), Table 4 (having accepted drug company sponsored products) and Table 5 (factors that influence the attitudes of students toward interactions between drug companies and medical students or physicians in logistic regression analysis).

**Table 3:** Effects of exposure to drug company representatives, exposure to drug company interactions during internship and prior rational prescription training on the attitudes of students towards interactions between drug companies and medical students or physicians at univariate analysis.

	Exposure to drug company represantatives		Exposure to drug company interactions during internship		Prior rational prescription training	
	Yes/No	P	Yes/No	P	Yes/No	P
**Influence of drug companies on prescription and perception of own knowledge of drugs**						
Interactions influence physicians’ prescription preferences (Disagreement or undecided)	29.0/40.0	0.385	26.0/52.0	**0.010**	27.6/35.4	0.329
I think that interactions influence resident physicians’ prescription preferences (Disagreement or undecided)	30.9/40.0	0.561	28.1/52.0	**0.019**	28.3/39.6	0.164
The interactions that I was exposed to may influence my future prescription preferences (Disagreement or undecided)	49.6/53.3	0.786	46.1/72.0	**0.018**	51.9/45.8	0.486
I possess sufficient knowledge about drugs to function as a general practitioner (Agreement)	30.2/33.3	0.775	32.0/24.0	0.426	28.3/35.4	0.374
My level of medical knowledge is sufficient to assess the information in drug advertisement brochures (Agreement)	61.6/46.7	0.262	65.4/36.0	**0.006**	61.0/58.3	0.759
**Skepticism towards accepting drug sponsored gifts and products**						
A public employee should never accept gifts (Agreement)	57.2/64.3	0.611	58.3/58.3	0.995	58.1/57.4	0.940
A medical student should never accept a gift from a drug company (Agreement)	32.4/40.0	0.572	32.0/40.0	0.439	32.1/35.4	0.683
There is nothing wrong in accepting small gifts as reminders, such as pens, key rings, memory sticks or bags (Disagreement or undecided)	43.9/33.3	0.433	44.5/36.0	0.431	45.3/37.5	0.366
A physician should not accept any gift from a drug company (Agreement)	41.0/46.7	0.673	43.0/36.0	0.518	43.4/37.5	0.492
I see nothing wrong in physicians attending scientific meetings sponsored by drug companies (Disagreement or undecided)	29.5/6.7	0.070	29.7/16.0	0.161	29.2/22.9	0.414
**Skepticism towards drug company–students–physician interactions other than accepting gifts**						
Interactions are an important source of information (Disagreement or undecided)	64.0/53.3	0.415	64.1/60.0	0.700	66.0/56.3	0.244
I trust the information in drug advertisement brochures (Disagreement or undecided)	83.9/73.3	0.290	83.3/84.0	1.000	87.5/72.9	**0.026**
The information provided during interactions is impartial (Agreement)	8.8/26.7	0.055	9.5/16.0	0.306	8.6/14.9	0.261
Interactions with students need to be subjected to legal regulation (Agreement)	73.9/73.3	1.000	75.0/70.8	0.668	79.2/61.7	**0.023**
I think that interactions with students are inadequate and need to be increased (Disagreement or undecided)	42.6/33.3	0.488	40.8/44.0	0.767	42.3/40.4	0.828
Drug companies should not hold activities in medical faculties (Agreement)	23.0/40.0	0.204	21.9/40.0	0.055	21.7/31.3	0.203
I regard interactions between pharmaceutical representatives and physicians as proper (Disagreement or undecided)	55.4/53.3	0.879	53.1/68.0	0.171	56.6/52.1	0.601
I am skeptical concerning the information provided by drug companies during interactions (Agreement)	81.3/78.6,	0.730	82.8/70.8	0.170	85.7/70.8,	**0.029**
I think that drug companies should support institutions rather than supporting physicians (Agreement)	47.8/60.0,	0.370	46.5/60.0	0.216	48.1/51.1,	0.736

**Table 4:** Effects of having accepted drug company sponsored products on the attitudes of students towards interactions between drug companies and medical students or physicians at univariate analysis.

	Accepted drug advertisement brochure		Accepted small, non-educational gifts		Accepted meal		Accepted free drug sample		Accepted textbook	
	At least once/Never	P	At least once/Never	P	At least once/Never	P	At least once/Never	P	At least once/Never	P
**Influence of drug companies on prescription and perception of own knowledge of drugs**										
Interactions influence physicians’ prescription preferences (Disagreement or undecided)	29.0/26.5	0.778	28.6/28.6	0.980	27.5/28.6	0.888	26.4/26.2	0.977	31.3/28.8	1.000
I think that interactions influence resident physicians’ prescription preferences (Disagreement or undecided)	30.6/35.3	0.605	30.3/32.1	0.845	25.0/36.9	0.149	25.9/33.3	0.356	31.3/32.8	0.903
The interactions that I was exposed to may influence my future prescription preferences (Disagreement or undecided)	48.1/58.8	0.278	48.7/53.6	0.645	44.2/56.0	0.184	46.3/51.2	0.575	50.0/51.3	0.925
I possess sufficient knowledge about drugs to function as a general practitioner (Agreement)	31.5/26.5	0.579	29.4/32.1	0.776	30.8/29.8	0.901	35.2/26.2	0.259	56.3/26.1	**0.019**
My level of medical knowledge is sufficient to assess the information in drug advertisement brochures (Agreement)	70.1/41.2	**0.002**	63.0/51.9	0.283	67.3/61.4	0.491	63.0/61.4	0.858	56.3/65.3	0.481
**Skepticism towards accepting drug sponsored gifts and products**										
A public employee should never accept gifts (Agreement)	54.2/64.7	0.282	57.1/53.6	0.732	59.6/54.8	0.579	59.3/57.1	0.806	56.3/57.1	0.946
A medical student should never accept a gift from a drug company (Agreement)	28.7/47.1	**0.047**	27.7/50.0	**0.023**	28.8/34.5	0.492	31.5/34.5	0.711	37.5/32.8	0.706
There is nothing wrong in accepting small gifts as reminders, such as pens, key rings, memory sticks or bags (Disagreement or undecided)	37.0/61.8	**0.011**	41.2/50.0	0.396	32.7/50.0	**0.048**	38.9/47.6	0.314	50.0/43.7	0.634
A physician should not accept any gift from a drug company (Agreement)	38.0/52.9	0.122	42.0/42.9	0.935	48.1/38.1	0.252	42.6/42.9	0.976	56.3/41.2	0.253
I see nothing wrong in physicians attending scientific meetings sponsored by drug companies (Disagreement or undecided)	25.9/32.4	0.464	26.1/35.7	0.305	17.3/33.3	**0.041**	27.8/29.8	0.802	31.3/26.9	0.767
**Skepticism towards drug company–students–physician interactions other than accepting gifts**										
Interactions are an important source of information (Disagreement or undecided)	65.7/61.8	0.672	63.0/67.9	0.632	65.4/63.1	0.787	72.2/59.5	0.128	56.3/64.7	0.509
I trust the information in drug advertisement brochures (Disagreement or undecided)	84.9/82.4	0.722	82.9/85.7	1.000	82.7/85.4	0.678	83.0/84.3	0.839	80.0/86.4	0.450
The information provided during interactions is impartial (Agreement)	8.5/17.6	0.133	9.3/14.3	0.489	9.8/11.9	0.706	9.4/11.9	0.652	6.3/10.2	1.000
Interactions with students need to be subjected to legal regulation (Agreement)	74.1/78.8	0.583	72.9/85.7	0.157	73.1/75.9	0.713	75.5/76.2	0.924	75.0/74.6	1.000
I think that interactions with students are inadequate and need to be increased (Disagreement or undecided)	37.1/55.9	0.054	43.6/35.7	0.448	44.0/41.7	0.792	36.5/46.4	0.257	43.8/41.9	0.887
Drug companies should not hold activities in medical faculties (Agreement)	20.4/38.2	**0.035**	21.8/35.7	0.125	21.2/28.6	0.336	29.6/22.6	0.356	25.0/26.1	1.000
I regard interactions between pharmaceutical representatives and physicians as proper (Disagreement or undecided)	50.9/70.6	**0.044**	52.9/60.7	0.457	42.3/60.7	**0.036**	50.0/57.1	0.411	37.5/57.1	0.138
I am skeptical concerning the information provided by drug companies during interactions (Agreement)	80.6/85.3	0.533	79.8/89.3	0.245	73.1/86.9	**0.043**	77.8/84.5	0.315	87.5/81.5	0.736
I think that drug companies should support institutions rather than supporting physicians (Agreement)	45.4/54.5	0.356	52.1/37.0	0.158	50.0/44.6	0.539	51.9/44.6	0.405	37.5/46.6	0.492

**Table 5:** Factors that influence the attitudes of students toward interactions between drug companies and medical students or physicians at logistic regression analysis.

	Time to graduation (months)	Related to or well acquainted with a pharmaceutical representative (Yes)	Planning a career in drug companies (Yes)	Exposure to drug company representatives (Yes)	Exposure to drug company interactions during internship (Yes)	Prior rational prescription training (Yes)	Accepted advertisement brochure (At least once)	Accepted small, non-educational gifts (At least once)	Accepted meal (At least once)	Accepted free drug sample(At least once)	Accepted textbook (At least once)
	Odds Ratio (95% C.I.)										
	P										
**Influence of drug companies on prescription and perception of own knowledge of drugs**											
Interactions influence physicians’ prescription preferences (Disagreement or undecided)	0.9(0.7–1.1)	1.3(0.4–3.6)	0.3(0.03–3.1)	9.9(0.8–120.9)	**0.2(0.05–0.9)**	0.4(0.2–1.2)	0.9(0.3–2.6)	1.2(0.4–3.8)	1.1(0.4–2.9)	0.9(0.3–2.6)	1.3(0.3–5.1)
	0.233	0.670	0.303	0.072	**0.039**	0.106	0.842	0.817	0.797	0.882	0.702
I think ^that^ interactions influence resident physicians’ prescription preferences (Disagreement or undecided)	1.1(0.9–1.3)	0.5(0.1–1.4)	1.7(0.3–10.5)	2.5(0.3–19.7)	0.6(0.1–2.2)	0.5(0.2–1.4)	0.9(0.3–2.5)	1.2(0.4–3.5)	0.6(0.3–1.6)	0.9(0.4–2.4)	1.5(0.4–5.5)
	0.497	0.180	0.574	0.377	0.393	0.190	0.849	0.762	0.313	0.897	0.520
The interactions that I was exposed to may influence my future prescription preferences (Disagreement or undecided)	0.9(0.7–1.1)	1.0(0.4–2.6)	0.6(0.1–4.0)	4.6(0.6–36.3)	**0.1(0.02–0.7)**	0.7(0.3–1.8)	0.8(0.3–2.1)	0.9(0.3–2.4)	0.7(0.3–1.7)	1.1(0.5–2.6)	1.2(0.4–4.2)
	0.153	0.962	0.576	0.150	**0.016**	0.454	0.690	0.824	0.457	0.846	0.743
I possess sufficient knowledge about drugs to function as a general practitioner (Agreement)	1.0(0.8–1.2)	0.7(0.2–2.0)	2.1(0.3–16.6)	0.2(0.03–1.9)	**7.1(1.1–45.5)**	0.5(0.2–1.4)	0.8(0.3–2.3)	0.6(0.2–2.0)	1.0(0.4–2.6)	**2.9(1.1–7.6)**	**5.5(1.5–19.8)**
	0.858	0.470	0.469	0.173	**0.038**	0.210	0.622	0.432	0.960	**0.033**	**0.009**
My level of medical knowledge is sufficient to assess the information in drug advertisement brochures (Agreement)	1.0(0.8–1.2)	1.3(0.5–3.8)	0.4(0.1–2.4)	0.7(0.1–5.0)	**4.6(1.1–19.1)**	0.8(0.3–2.2)	**4.9(1.8–13.6)**	1.2(0.4–3.4)	0.7(0.3–1.8)	1.4(0.5–3.7)	0.5(0.1–1.7)
	0.759	0.603	0.293	0.723	**0.038**	0.645	**0.002**	0.790	0.477	0.533	0.256
**Skepticism towards accepting drug sponsored gifts and products**											
A public employee should never accept gifts (Agreement)	1.1(0.9–1.4)	1.9(0.7–4.9)	0.4(0.1–2.8)	0.7(0.1–3.8)	1.1(0.3–4.3)	1.2(0.5–3.0)	0.5(0.2–1.3)	1.8(0.6–4.9)	1.4(0.6–3.2)	0.7(0.3–1.7)	0.9(0.3–3.0)
	0.264	0.178	0.384	0.632	0.880	0.718	0.151	0.282	0.461	0.448	0.866
A medical student should never accept a gift from a drug company (Agreement)	1.1(0.9-.1.3)	1.0(0.4–2.6)	1.5(0.2–9.8)	1.2(0.2–7.8)	0.9(0.2–3.6)	0.7(0.3–1.8)	0.4(0.2–1.1)	0.5(0.2–1.5)	1.2(0.5–2.9)	0.9(0.3–2.3)	1.7(0.5–5.9)
	0.502	0.937	0.653	0.829	0.863	0.426	0.086	0.230	0.748	0.802	0.415
There is nothing wrong in accepting small gifts as reminders, such as pens, key rings, memory sticks or bags (Disagreement or undecided)	1.1(0.9–1.3)	0.9(0.3–2.2)	1.0(0.2–6.4)	2.4(0.3–1.6.6)	1.8(0.4–7.7)	1.1(0.4–2.8)	0.4(0.2–1.0)	0.9(0.3–2.5)	0.7(0.3–1.6)	0.8(0.3–1.8)	1.9(0.6–6.2)
	0.458	0.737	0.998	0.387	0.405	0.862	0.057	0.864	0.378	0.545	0.311
A physician should not accept any gift from a drug company (Agreement)	0.9(0.7–1.1)	0.8(0.3–2.0)	0.3(0.04–3.4)	0.5(0.1–2.8)	1.2(0.3–5.2)	1.1(0.4–2.8)	**0.3(0.1–0.9)**	1.7(0.6–4.8)	2.0(0.8–4.7)	1.0(0.4–2.4)	1.8(0.5–5.8)
	0.326	0.631	0.363	0.418	0.782	0.910	**0.030**	0.334	0.125	0.953	0.351
0.6(0.2–1.7)	0.9(0.7–1.2)		0.5(0.04–5.6)	8.8E8	0.9(0.2–4.3)	0.9(0.3–2.7)	1.0(0.4–2.8)	0.6(0.2–1.7)	0.4(0.1–1.0)	1.1(0.4–3.0)	1.3(0.3–5.3)
	0.442	0.306	0.592	0.999	0.861	0.864	0.985	0.337	0.050	0.839	0.672
**Skepticism towards drug company–students–physician interactions other than accepting gifts**											
Interactions are an important source of information (Disagreement or undecided)	1.0(0.8–1.2)	0.9(0.4–2.4)	0.6(1.0–3.5)	1.0(0.2–5.8)	0.7(0.2–2.8)	1.6(0.7–4.1)	1.0(0.4–2.7)	0.7(0.3–2.0)	1.2(0.5–2.8)	1.7(0.7–4.1)	0.5(0.2–1.7)
	0.911	0.869	0.554	0.992	0.616	0.290	0.923	0.547	0.654	0.244	0.288
I trust the information in drug advertisement brochures (Disagreement or undecided)	1.0(0.8–1.3)	0.7(0.2–2.5)	2.7E8	4.9(0.6–40.0)	0.4(0.04–3.2)	2.0(0.6–6.6)	1.3(0.3–4.6)	0.8(0.2–3.4)	1.0(0.3–3.3)	1.1(0.3–3.8)	0.8(0.1–4.0)
	0.989	0.624	0.999	0.136	0.351	0.260	0.718	0.776	0.984	0.826	0.762
The information provided during interactions is impartial (Agreement)	0.8(0.5–1.2)	0.3(0.02–3.8)	2.9(0.2–38.4)	0.1(0.01-.1.3)	1.1(0.1–11.1)	0.3(0.1–1.4)	0.3(0.1–1.4)	2.1(0.3–14.6)	1.0(0.2–5.1)	0.8(0.2–4.2)	0.8(0.8–11.5)
	0.205	0.364	0.410	0.085	0.929	0.121	0.109	0.460	0.977	0.801	0.841
Interactions with students need to be subjected to legal regulation (Agreement)	1.0(0.8–1.2)	0.7(0.3–2.2)	0.3(0.04–2.4)	1.3E-9	0.1(0.01–1.6)	**3.5(1.2–10.2)**	1.3(0.4–4.2)	0.4(0.1–1.7)	0.8(0.3–2.1)	0.8(0.3–2.3)	0.4(0.1–1.7)
	0.801	0.596	0.255	0.999	0.112	**0.021**	0.662	0.216	0.668	0.736	0.211
I think that interactions with students are inadequate and need to be increased (Disagreement or undecided)	0.9(0.8–1.1)	0.7(0.3–1.9)	1.1(0.2–6.2)	2.5(0.4–14.6)	0.5(0.1–1.7)	0.8(0.3–2.0)	**0.3(0.1–0.9)**	2.5(0.9–7.4)	1.8(0.8–4.2)	0.6(0.2–1.4)	1.6(0.5–5.3)
	0.496	0.488	0.952	0.312	0.252	0.630	**0.028**	0.091	0.188	0.241	
Drug companies should not hold activities in medical faculties (Agreement)	1.1(0.9–1.3)	1.1(0.4–3.1)	0.7(0.1–7.0)	0.6(0.1–3.5)	0.7(0.2–3.0)	0.4(0.2–1.2)	0.5(0.2–1.4)	0.4(0.1–1.4)	0.9(0.3–2.4)	2.2(0.8–6.0)	1.2(0.3–4.9)
	0.499	0.925	0.752	0.528	0.638	0.115	0.197	0.171	0.773	0.137	0.788
I regard interactions between pharmaceutical representatives and physicians as proper (Disagreement or undecided)	0.8(0.7–1.0)	2.4(0.9–6.4)	1.6(0.2–11.6)	3.9(0.5–28.2)	0.2(0.1–1.1)	1.1(0.4–2.9)	0.4(0.2–1.2)	1.5(0.5–4.5)	0.6(0.2–1.4)	0.6(0.3–1.5)	0.4(0.1–1.6)
	0.081	0.093	0.617	0.182	0.059	0.836	0.110	0.429	0.223	0.306	0.205
I am skeptical concerning the information provided by drug companies during interactions (Agreement)	1.0(0.8–1.3)	1.3(0.4–4.6)	0.6(0.1–4.1)	0.4(0.02–4.3)	1.1(0.2–5.8)	**3.7(1.2–11.5)**	1.3(0.3–4.8)	1.0(0.2–4.7)	**0.3(0.1–0.97)**	0.7(0.2–2.2)	1.1(0.2–5.9)
	0.946	0.718	0.596	0.413	0.903	**0.022**	0.744	0.974	**0.043**	0.523	0.929
I think that drug companies should support institutions rather than supporting physicians (Agreement)	1.0(0.8–1.3)	**3.3(1.2–8.7)**	0.2(0.01–1.7)	0.6(0.1–3.6)	0.3(0.1–1.4)	1.2(0.5–3.1)	0.5(0.2–1.4)	2.6(0.8–7.8)	1.4(0.6–3.3)	0.8(0.3–1.9)	0.4(0.1–1.5)
	0.816	**0.016**	0.124	0.581	0.122	0.727	0.196	0.102	0.486	0.563	0.159

### a. Exposure of students to drug company representatives

Exposure to drug company representatives was not significantly associated with any attitude on univariate and logistic regression analysis (p>0.05 for all).

### b. Exposure of students during internship

Exposure to drug company interactions during internship was determined to significantly affect all propositions concerning the influence of drug companies on prescription writing and subjects’ perception of their knowledge of drugs. Students who had been exposed at least once expressed significantly greater self-confidence concerning their knowledge of drugs. Exposure during internship was not found to be associated with attitudes related to skepticism.

### c. Prior rational prescription training

Prior rational prescription training was not determined to significantly affect attitudes concerning the influence of drug companies on prescription writing and subjects’ perception of their knowledge of drugs and skepticism concerning accepting gifts and drug company sponsored products. In comparison with students who had received training in rational drug prescribing, those who had not expressed a significantly higher degree of disagreement/indecision with the proposition “I trust the information in drug advertisement brochures” (87.5 vs 72.9%, p = 0.026); expressed greater agreement with the statement “Interactions with students need to be subjected to legal regulation” [O.R.(95% C.I.)][3.5(1.2–10.2), p = 0.021]; and expressed greater agreement with the statement “I am skeptical concerning the information provided by drug companies during interactions”, and this finding was supported by logistic regression [3.7(1.2–11.5), p = 0.022].

### d. Acceptance of drug company sponsored gifts and products (drug advertisement brochures, small, non-educational gifts, meals, free drug samples, textbooks)

Students who had accepted drug advertisement brochures at least once expressed significantly higher agreement with the statement “My level of medical knowledge is sufficient to assess the information in drug advertisement brochures” than students who had never accepted them (4.9[1.8–13.6], p = 0.002). Students who had accepted textbooks (5.5[1.5–19.8], p = 0.009) and free drug samples (2.9[1.1–7.6], p = 0.033) from drug companies expressed a high level of agreement with the proposition “I possess sufficient knowledge about drugs to function as a general practitioner”. Accepting advertisement brochures was found to significantly reduce agreement with the proposition that “A physician should not accept any gift from a drug company” (0.3[0.1–0.9], p = 0.030) and disagreement with the proposition “I think that interactions with students are inadequate and need to be increased” (0.3[0.1–0.9], p = 0.028). Accepting meals significantly reduced agreement with the proposition “I am skeptical concerning the information provided by drug companies during interactions” (0.3[0.1–0.97], p = 0.043).

## Discussion

### 1. Exposure of students to drug company interactions and the extent of the exposure

There are almost no restrictions on interactions between drug companies and students in our university. Interactions with pharmaceutical representatives may take place anywhere in our medical school and its clinical areas, from lecture rooms to clinics. Our results show a high rate of Turkish student exposure to drug companies and interactions with drug company representatives, consistent with the findings in numerous previous studies in many countries worldwide [1, 11, 17, 19, 28]. Almost all students reported exposure to drug companies, as was also reported by Bellin et al. for the University of Minnesota in 2001 [28] and by Sierles et al. for 8 U.S. medical schools in 2003 [11]. Austad et al. reported that 40% to 100% of medical students had experienced an interaction with a drug company in their systematic review [19]. In agreement with the literature [1, 17, 28], attendance begins with the first year and intensifies with clinical training, as expected.

Students in our study stated that they frequently accepted gifts, similarly to other medical students. The level of acceptance of small, non-educational gifts was similar to those of residents in France and Germany and medical students in the USA, but lower compared to medical students in Kuwait. The level of acceptance of meals was similar to that for accepting gifts [11, 21, 29].

### 2. Attitudes of students towards interactions between drug companies and medical students or physicians and related factors

#### a. Influence of drug companies on prescription and perception of own knowledge of drugs

Our students were disposed to feel that interactions may influence physicians’ prescription-writing in general, but felt that individually they would be less influenced. This is similar to the findings from studies of students and physicians from both developed and developing countries [1, 23, 30], and is also in agreement with findings concerning pre-clinical students in Turkey [27]. In addition, the idea that their own prescription writing might be affected was more common among students who think that drug company interactions affect physicians. At this point, we observed that exposure to drug company interactions during internship was significantly related to the idea that drug company interactions influence prescription behaviors. This finding may be due to students spending an increasing amount of time at the clinic and thus having a better opportunity to observe the impacts of drug companies. Further evidence to support the idea that the longer students work in a clinic the more they are influenced appeared in a study performed among pre-clinical students in Turkey by Sarıkaya et. al. In that study, 55.3% of students stated that physicians would be affected by gifts from drug companies and that there would be a greater probability of their prescribing drugs made by gift-giving companies [27]. Similarly, 68.2% of the students in our study thought that interactions influence resident physicians’ prescription preferences. Since we observed that exposure during internship affects the perception of the influence of drug companies on physicians’ prescription writing habits, we think that the scope of the directive issued for physicians [18] should therefore be broadened to include medical students.

#### b. Skepticism

In addition to the high prevalence of exposure to drug company interactions among the students in our study, our students tend to have both permissive and intolerant attitudes, but a much higher level of permissive attitudes toward interactions between students and drug companies. Our students thought that drug company–student interactions were a proper and important source of information, and there was a demand for more interactions because 58.3% of them thought that the number of interactions with students is inadequate and needs to be increased (Table 2). Students were permissive toward accepting gifts and attending dug company sponsored scientific meetings. They were largely against permitting dug company activities in medical faculties. They also commonly reported being skeptical concerning information provided by drug companies and not trusting the information in drug advertisement brochures. In their comprehensive systematic review, Austad KE at al. reported that students’ attitudes were variable, being permissive in terms of accepting meals or small promotional items, but being opposed to travel and social events [19]. Our students also welcoming the idea of travel, but only for scientific purposes. Attitudes regarding accepting gifts in particular lie somewhere between those determined in previous studies by Sierles in 2003 and 2012 [31]. Univariate analyses concerning accepting small, non-educational gifts revealed that accepting a gift at least once reduced agreement with the proposition “A medical student should never accept a gift from drug companies” from 50% to 27.7% (p = 0.023) (Table 4).

Advertisement brochures were found to be effective both in univariate and regression analysis in creating a permissive approach. This finding is also relevant to the findings of Alssageer et al. in Libya, who reported that 75% of physicians support accepting gifts from drug companies and that physicians who had previously accepted gifts had a greater tendency to accept them again [23]. Additionally, accepting drug company sponsored meals significantly reduced agreement with the idea that a physician should not accept any gift from a drug company in logistic regression. When these findings are considered together, it appears that direct exposure (accepting advertisement brochures, gifts or meals) to drug companies increased positive perceptions towards such companies on the part of students. This opinion was also expressed in Sarıkaya’s qualitative study assessing the views of pre-clinical medical students in Turkey [27]. Providing gifts therefore seems to represent a way of reducing intolerant attitudes towards drug companies, and prohibiting the acceptance of certain gifts by medical students might therefore be considered for inclusion in policies concerning drug company–medical student interactions.

Drug company-physician and drug company-student interactions, and permissive attitudes regarding these, are associated with presentation of information that is biased in favor of company-sponsored products, leading to non-rational prescription choices involving products with no significant superiority over others, more expensive drugs and fewer generic drugs [6]. Although information may be missing from advertising brochures, physicians have reported that these affect their prescription preferences and are commonly used as advertising tools by drug companies [32, 33]. Our study elicited some interesting results concerning such brochures. Those students who had taken a drug advertising brochure at least once tended to express less agreement with the proposition that medical students should never accept gifts, as discussed above. These students were also permissive in terms of accepting small gifts in univariate analysis and desired an increase in interactions with drug companies in regression analysis. These two findings show that drug advertising brochures may lead to more permissive attitudes among medical students. Besides these effects of drug advertisement brochures, interestingly most of our students stated that they did not trust the information provided in drug advertisement brochures but accepting brochures was not related to this attitude. This may show that the students were skeptical about the information in advertisement brochures but were not against accepting them. This is also similar to the finding that we discussed above about the influence of drug companies on prescription habits whereby students stated they felt that physicians are generally affected but that individually they will not be. They may also think that “I will not be affected and therefore I can accept the advertisement brochure”, but we found that accepting brochures affects their attitudes towards accepting gifts. A more in depth analysis of these variable attitudes with a qualitative study will be useful in understanding underlying causes. Additionally, the only variable that affects the attitude about trusting information in drug advertisement brochures was receiving rational prescription training.

#### c. Rational prescription training

This study elicited interesting results concerning the effect of rational prescription training. Such training significantly increased the demand for legal regulations regarding interactions between students and drug companies. Additionally, students who had received such training were significantly more skeptical concerning information provided by drug companies. These two findings were constant in both univariate and regression analysis. Students who had received such training also placed less trust in the information in advertisement brochures. These results show that rational prescription training raises students’ awareness of the need for a more skeptical attitude toward information or potentially misleading information in such brochures [32]. Interestingly, rational prescription training was not observed to be effective in developing intolerant attitudes regarding accepting gifts. This may due to the content of the training, since it does not include issues such as advertising tactics or exposure to drug companies and their potential effects. Teaching about drug company-student and drug company-physician interactions can help increase healthy skeptical attitudes in this area [34].

### Limitations

Our study has some limitations. It was retrospective, and students’ recollections—like those of any survey respondents, were necessarily imperfect. The recall bias might be greater for the questions about the first exposure time, the first exposure site and types of exposures. In addition, although our findings replicate those from many other international studies, our study’s findings represent the results from a single university and therefore a relatively low number of students could be included.

In conclusion, the interactions between final year medical students and drug companies are common. These interactions may affect the students’ attitudes. Our results suggest that rational prescription training and similar educational activities may create sensitivity and awareness concerning the possible influences of drug companies on medical students and may contribute to students developing a healthy skepticism. We recommend that Turkish governmental and academic regulatory agencies establish or recommend policies regulating drug company-student interaction, and require or recommend teaching and learning about drug company-student and drug company-physician interactions [35–37]. There is evidence that developing such policies leads to less frequent industry contacts or to more skeptical attitudes [10]. In addition, regulations limiting interactions are not by themselves sufficient, and instruction concerning interactions between drug companies and medical students should also be provided. One example of such a teaching and learning process is the rational prescription training that we provide for our students. An examination of other factors (e.g., media attention, scientific publication, litigation) that might reduce medical student and physician exposure to drug company interactions and increase healthy skepticism about such interactions in Turkey and the rest of the developing and developed world is beyond the scope of this investigation.

## Supporting Information

S1 Fig Questionnaire. (DOCX)

S2 Fig Data. (XLSX)
