# Supplementary material for: Turkish Final Year Medical Students’ Exposure to and Attitudes Concerning Drug Company Interactions: A Perspective from a Minimally Regulated Environment for Medical Students
Source: PLoS One. 2016 Dec 15;11(12):e0168094. doi: 10.1371/journal.pone.0168094 (PMC5158011; doi:10.1371/journal.pone.0168094)
Supplement: S1 Fig — (DOCX) [file pone.0168094.s001.docx]

**TURKISH MEDICAL INTERNS EXPOSURE TO AND ATTITUDES ABOUT DRUG COMPANY INTERACTIONS**

| This study aims to determine your, namely the interns’, condition of participation to drug company interactions and attitude towards these interactions. Participation to this survey is carried out on voluntary basis. Your participation will be regarded as consent. Your replies to the questions will not be used for any other intentions but only for scientific purposes. Please, do not write any information that may hint your name or identity on the survey. Thanks for your interest.    **KTU Faculty of Medicine Department of Public Health( tel: 3775122)** |
| --- |

**SOCİODEMOGRAPHIC AND PERSONAL FEATURES**

**1. Your birth year :**.................. (please write)

**2. Gender :** ( ) Male ( ) Female

**3. Do you know any pharmaceutical representative among your family members or close acquaintances?**

( ) No ( ) Yes

**4. How much time left have you got to graduate?** ............. months (please write)

**QUESTIONS ABOUT DRUG COMPANY INTERACTIONS**

**5. Have you ever been trained on rational drug prescription during your medical education?**

( ) No ( ) Yes

**6. Have you ever been exposed to an interaction with a pharmaceutical representative of a drug company?( you may mark more than one option)**

( ) No, never

( ) Yes / One-to-one

( ) Yes / In a student group

( ) Yes / Alongside a resident

( ) Yes / Alongside an instructor physician

( ) Yes / Other ……………….(please write)

**7. How often have you participated to drug company interactions during your internship?**

( ) A couple of times a week

( ) Once a week

( ) A couple of times a month

( ) Once a month

( ) More rare than once a month

( ) Never participated

**8. What grade were you when you first exposed to a drug company interaction?**

( ) 1 ( ) 2 ( ) 3 ( ) 4 ( ) 5 ( ) 6

**9. Where was the drug company interaction that you first exposed held?**

( ) In a lecture room

( ) At a scientific congress

( ) At a meal the company organized

( ) At an outpatient clinic during clinical education period

( ) At an inpatient clinic during clinical education period

( ) Other ……………….. (please write)

**10. Please mark (X) how often you have accepted any drug company promotional products during your medical education**

|  | Never | Once | 2to5 times | More than 5 |
| --- | --- | --- | --- | --- |
| 1. Drug advertisement brochures |  |  |  |  |
| 2. Non-educational small gifts (pen, notebook, stationery materials, calender, USB flashdisc, bag, etc.) |  |  |  |  |
| 3. Free drug sample |  |  |  |  |
| 4. Textbook |  |  |  |  |
| 5. Sponsored travel, conference, congress |  |  |  |  |
| 6. Meals |  |  |  |  |
| 7. other …………… (please write) |  |  |  |  |

**11.Do you think of planning a carrier at a drug company?**

( ) no ( ) yes

**PROPOSITIONS**

**There are some propositions below. Mark (X) your decisions towards these propositions.**

|  |  | **Strongly disagree** | **Disagree** | **Undecided** | **Agree** | **Strongy agree** |
| --- | --- | --- | --- | --- | --- | --- |
| P1 | I possess sufficient knowledge about drugs to function as a general practitioner. |  |  |  |  |  |
| P2 | Interactions are an important source of information. |  |  |  |  |  |
| P3 | I trust the information in drug advertisement brochures. |  |  |  |  |  |
| P4 | The information provided during interactions is impartial. |  |  |  |  |  |
| P5 | Interactions influence physicians’ prescription preferences. |  |  |  |  |  |
| P6 | A public employee should never accept gifts. |  |  |  |  |  |
| P7 | I think that interactions influence resident physicians’ prescription preferences. |  |  |  |  |  |
| P8 | Interactions with students need to be subjected to legal regulation. |  |  |  |  |  |
| P9 | A medical student should never accept a gift from a drug company. |  |  |  |  |  |
| P10 | I think that interactions with students are inadequate and need to be increased. |  |  |  |  |  |
| P11 | There is nothing wrong in accepting small gifts as reminders, such as pens, key rings, memory sticks or bags. |  |  |  |  |  |
| P12 | Drug companies should not hold activities in medical faculties. |  |  |  |  |  |
| P13 | A physician should not accept any gift from a drug company. |  |  |  |  |  |
| P14 | My level of medical knowledge is sufficient to assess the information in drug advertisement brochures. |  |  |  |  |  |
| P15 | I see nothing wrong in physicians attending scientific meetings sponsored by drug companies. |  |  |  |  |  |
| P16 | The interactions that I was exposed to may influence my future prescription preferences. |  |  |  |  |  |
| P17 | I regard interactions between pharmaceutical representatives and physicians as proper. |  |  |  |  |  |
| P18 | I am skeptical concerning the information provided by drug companies during interactions. |  |  |  |  |  |
| P19 | I think that drug companies should support institutions rather than supporting physicians. |  |  |  |  |  |
